# On the Durability of Tin‐Containing Perovskite Solar Cells

**DOI:** 10.1002/advs.202304811

**Published:** 2023-11-15

**Authors:** Lei Chen, Sheng Fu, You Li, Nannan Sun, Yanfa Yan, Zhaoning Song

**Affiliations:** ^1^ Department of Physics and Astronomy and Wright Center for Photovoltaics Innovation and Commercialization The University of Toledo 2801 W. Bancroft Street Toledo OH 43606 USA

**Keywords:** degradation mechanisms, mitigation strategies, perovskite solar cells, stability, tin perovskites

## Abstract

Tin (Sn)‐containing perovskite solar cells (PSCs) have gained significant attention in the field of perovskite optoelectronics due to lower toxicity than their lead‐based counterparts and their potential for tandem applications. However, the lack of stability is a major concern that hampers their development. To achieve the long‐term stability of Sn‐containing PSCs, it is crucial to have a clear and comprehensive understanding of the degradation mechanisms of Sn‐containing perovskites and develop mitigation strategies. This review provides a compendious overview of degradation pathways observed in Sn‐containing perovskites, attributing to intrinsic factors related to the materials themselves and environmental factors such as light, heat, moisture, oxygen, and their combined effects. The impact of interface and electrode materials on the stability of Sn‐containing PSCs is also discussed. Additionally, various strategies to mitigate the instability issue of Sn‐containing PSCs are summarized. Lastly, the challenges and prospects for achieving durable Sn‐containing PSCs are presented.

## Graphical abstract

This review summarizes the intrinsic and extrinsic degradation pathways of tin‐containing perovskite solar cells (PSCs) and discusses mitigation strategies to enhance their durability. Prospectives on potential avenues for advancing tin‐containing PSCs are also presented.

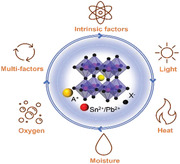

## 1 Introduction

In recent years, the remarkable progress of photovoltaic (PV) performance exhibited by metal halide perovskite solar cells (PSCs) has garnered considerable attention, underscoring their immense potential for next‐generation renewable power generation. This emerging PV technology is based on a family of metal‐halide perovskite materials (ABX_3_), which consist of various organic and inorganic monovalent A cations, such as methylammonium (MA), formamidinium (FA), and cesium (Cs), divalent metal B cations, such as lead (Pb), tin (Sn), and germanium (Ge), and halide X anions, including iodine (I), bromine (Br), and chlorine (Cl) mixtures in their lattice, where the Goldschmidt tolerance factor generally governs the formation of the 3D perovskite phase.^[^ ^1^, ^2^, ^3^ ^]^

PSCs have witnessed a noteworthy advancement in power conversion efficiency (PCE), surging from a modest 3.8% to a certified 26.1% in the past decade.^[^ ^4^, ^5^, ^6^, ^7^, ^8^ ^]^ Notwithstanding the commendable advancements achieved in the Pb‐based PSCs, the inherent toxicity of Pb raises valid apprehensions regarding their potential environmental and health ramifications. In scenarios where Pb‐based perovskites encounter environmental exposures, such as device encapsulation failures and landfills as they reach the end of their life cycles, there exists the possibility of Pb^2+^ leakage into soil and water.^[^ ^9^, ^10^, ^11^, ^12^ ^]^

In the pursuit of finding a suitable substitute for Pb in PSCs, Sn‐based perovskites have emerged as a primary and highly promising candidate.^[^ ^13^, ^14^ ^]^ This is primarily attributed to Sn's significantly lower toxicity levels observed across various testing conditions, making it a favorable alternative to Pb.^[^ ^15^ ^]^ Similar to Pb‐based perovskites, Sn‐containing perovskites, including Sn and mixed Sn‐Pb perovskites, exhibit high charge carrier mobility and a small exciton binding energy.^[^ ^16^, ^17^, ^18^ ^]^ Moreover, manipulating the Sn/Pb ratio within Sn‐containing perovskite materials leads to bandgap modulation within the range of 1.2 to 1.5 eV.^[^ ^19^, ^20^ ^]^ This particular bandgap range is appealing in solar cell applications because it can effectively harness the solar radiation spectrum to achieve the maximum theoretical efficiency limits of a single‐junction solar cell.^[^ ^21^ ^]^ Additionally, the narrow‐bandgap Sn‐Pb perovskites can be paired with wide‐bandgap perovskite to fabricate ultrahigh efficiency all‐perovskite tandem solar cells.^[^ ^22^, ^23^, ^24^ ^]^

Nevertheless, the device efficiency and stability of Sn‐containing PSCs significantly lag behind their Pb‐based counterparts.^[^ ^25^, ^26^, ^27^, ^28^ ^]^ This disparity is attributed to the instability of Sn^2+^ species within the perovskite structure,^[^ ^29^ ^]^ which exhibits inherent instability, rendering them susceptible to conversion into Sn^4+^ products at various stages throughout the lifespan of the material. Notably, this conversion process can occur even in the precursor solution with mild oxidants, including DMSO.^[^ ^30^, ^31^ ^]^ Due to Sn^2+^ oxidation, Sn‐containing perovskite materials display high p‐type doping characteristics, leading to a shortened carrier lifetime detrimental to the overall device performance.^[^ ^32^ ^]^ Moreover, the degradation products, such as SnI_4_ and I_2_, can actively participate in and catalyze the degradation process, consequently impacting the stability of Sn‐containing PSCs.^[^ ^33^ ^]^ Over the years, considerable effort has been made to understand the unique degradation mechanisms of Sn‐containing perovskites and develop accordingly appropriate mitigation strategies.^[^ ^34^, ^35^, ^36^ ^]^ Still, more comprehensive insights on the durability of Sn‐containing perovskites are needed to guide the future development of these new materials and fully realize their potential.

In this review, we present a compendious overview of the degradation mechanisms associated with Sn‐containing perovskites and strategies to enhance their stability. First, an in‐depth analysis of the degradation pathways of Sn‐containing perovskites is summarized, encompassing the intrinsic durability of the materials, extrinsic stability under external stresses, such as light, heat, moisture, oxygen, their synergistic effects, and the impact of interface and electrode materials. Next, various advanced strategies to address the instability issues of Sn‐containing perovskites are highlighted, including implementing antioxidant and reducing agents, compositional engineering, interface and grain boundary passivation, and encapsulation engineering. Lastly, insightful perspectives regarding future advancements and potential directions for developing Sn‐containing perovskites are provided.

## 2 Degradation Mechanisms of Sn‐Containing PSCs

### 2.1 Intrinsic Degradation Pathways

#### 2.1.1 Intrinsic Material Stability

**Figure 1:**
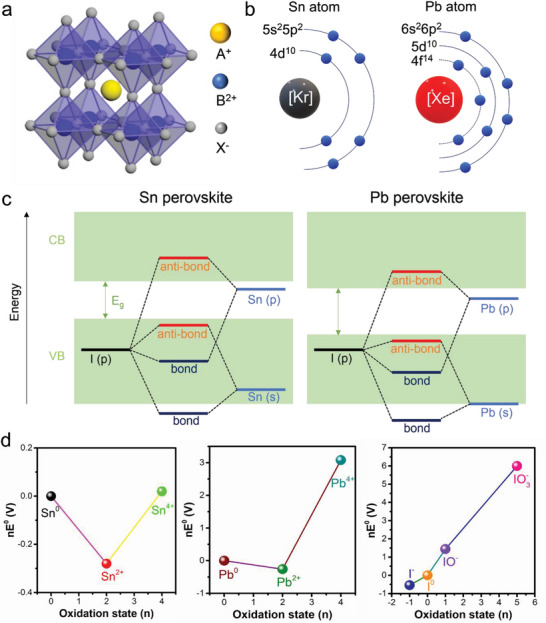
a) Schematic diagram of the perovskite structure. b) Schematic illustration of the external electron configuration of Sn and Pb atoms. Reproduced with permission.^[^ ^37^ ^]^ Copyright 2021, American Chemical Society. c) Schematic diagram of the band structure of Sn and Pb perovskites. Reproduced with permission.^[^ ^129^ ^]^ Copyright 2018, American Chemical Society. d) Frost diagram of Sn and Pb in standard conditions and I in acidic conditions. Reproduced with permission.^[^ ^37^ ^]^ Copyright 2021, American Chemical Society.

Group IV‐A heavy elements Pb and Sn possess the +2 oxidation state due to the inert‐part effect, making them suitable for the ABX_3_ perovskite crystal structure (**Figure** **1**a). However, Sn‐containing perovskites exhibit inferior stability than Pb‐perovskites due to the ease of Sn^2+^ oxidation to Sn^4+^. This easy oxidation is related mainly to the electron configuration of Sn, which is [Kr] 4d^10^5s^2^5p^2^ (Figure 1b). Due to the absence of lanthanide contraction, Sn's valence electrons in 5s and 5p orbitals are easy to lose, leading to the formation of Sn^4+^.^[^ ^37^ ^]^ In contrast, Pb atom has an electron configuration of [Xe] 4f^14^5d^10^6s^2^6p^2^. A strong nuclear attraction exerted on the 6s electrons due to the weak electron shielding effect of the intervening 4f and 5d orbitals hinders the removal of the long pair 6s electrons, resulting in the stable +2 oxidation state of Pb.

Apart from the electron configuration, the facile oxidation of Sn is attributed to the band structure of Sn‐containing perovskite (Figure 1c). In metal‐halide perovskites, the conduction band minimum (CBM) is predominantly determined by the p orbitals of metal atoms, while the valence band maximum (VBM) is composed of the antibonding coupling between the s orbitals of metal atoms and the p orbitals of halide atoms (e.g., I). Due to higher energy in the Sn 5s orbital than that in the Pb 6s orbital, the anti‐bond of Sn‐I is less stable and energetically more favorable to break than the Pb–I bond, resulting in a higher density of Sn vacancies in Sn‐containing perovskites.^[^ ^38^ ^]^ This provides compelling evidence for the intrinsic propensity of Sn^2+^ to be oxidized to Sn^4+^ in Sn‐containing perovskites.

Beyond the energy band levels, the electrochemical stability of Sn‐perovskite also presents a significant challenge. Figure 1d plots the Frost–Ebsworth diagrams of different oxidation states of Sn, Pb, and I, which are calculated based on their standard electrode potentials as follows:

(1) $${\mathrm{Sn}}^{4+}+2e^{-}\to {\mathrm{Sn}}^{2+}\;E^{0}=+0.15\;V$$

(2) $${\mathrm{Sn}}^{2+}+2e^{-}\to {\mathrm{Sn}}^{0}\;E^{0}=-0.13\;V$$

(3) $${\mathrm{Pb}}^{4+}+2e^{-}\to {\mathrm{Pb}}^{2+}\;E^{0}=+1.67\;V$$

(4) $${\mathrm{Pb}}^{2+}+2e^{-}\to {\mathrm{Pb}}^{0}\;E^{0}=-0.13\;V$$

(5) $$I_{2}+2e^{-}\to 2I^{-}\;E^{0}=+0.54\;V$$

(6) $$I_{3}^{-}+2e^{-}\to 3I^{-}\;E^{0}=+0.53\;V$$

The standard redox potential of Sn^4+^/Sn^2+^ is +0.15 V, indicating significantly lower thermodynamic stability than that of Pb^4+^/Pb^2+^ with a redox potential of +1.67 V.^[^ ^39^ ^]^ This suggests that Sn^2+^ is prone to be oxidized. Therefore, the fabrication conditions of Sn‐containing perovskite should be meticulously controlled to prevent oxidation. However, despite careful control of the fabrication environment, the complete exclusion of oxygen exposure is challenging, and partial oxidation of Sn^2+^ source materials is likely to occur during the preparation process, which provides a pathway for the decomposition of Sn‐containing perovskites.^[^ ^40^ ^]^ In addition, it is noteworthy that the redox potential of I_2_/I^−^ is +0.54 V, which is close to or lower than typical quasi‐Fermi energy splitting between two electrodes and the operating voltages observed in PSCs. As a result, the possibility exists for oxidation of I^−^ to I_2_ within PSCs when subjected to an applied voltage or under an illumination bias.^[^ ^41^, ^42^ ^]^ Moreover, the redox potential of I_2_/I^−^ (+0.54 V) is larger than that of Sn^4+^/Sn^2+^ (+0.15 V), indicating that the released I_2_ is thermodynamically favorable to subsequently oxidize free Sn^2+^ to Sn^4+^,^[^ ^43^ ^]^ leading to further degradation of Sn‐containing perovskite even under an inert environment.

#### 2.1.2 Surface Defects

Surfaces, grain boundaries, and interfaces are crucial factors controlling the optoelectronic properties and stability of Sn‐containing perovskites. Surface deterioration and the oxidation process of Sn^2+^ ions are more likely to occur at the surfaces of Sn‐containing perovskites than the bulk material because of high‐density undercoordinated ions on defective surfaces.^[^ ^44^ ^]^ Specifically, the undercoordinated Sn^2+^ ions on the surface are highly susceptible to adsorb oxygen or iodine molecules, leading to their rapid oxidation and the generation of Sn^4+^ defects. Additionally, surface Sn^2+^ ions are also prone to undergo disproportionation, resulting in the creation of Sn vacancies, as depicted by the following equation:

(7) $$2{\mathrm{Sn}}^{2+}\to {\mathrm{Sn}}^{4+}+{V_{\mathrm{Sn}}}^{2-}+2h^{+}+{\mathrm{Sn}}^{0}$$

The presence of these surface Sn^4+^ defects, which act as deep electron traps, exacerbates the irreversible degradation of the perovskite material, leading to the formation of secondary phases such as FA_2_SnI_6_ and SnI_4_.^[^ ^33^ ^]^ This degradation process significantly compromises the long‐term stability of Sn‐containing PSCs.

Apart from the surface defects associated with Sn, it is important to consider other native surface defects, such as I vacancies, as they are likely to have a significant impact on the degradation process of perovskite materials. These I vacancies are believed to facilitate the migration of iodide ions and promote the formation of I_2_/I_3_^−^ species, which are known to contribute to degradation.^[^ ^45^ ^]^ It is plausible that the appearance of I vacancies may commence perovskite degradation, particularly under ambient conditions. This behavior is analogous to the degradation mechanism observed in Pb‐based perovskites. In the presence of oxygen and illumination, I vacancies have been experimentally shown to attract aggressive superoxide species from atmospheric oxygen preferentially. These superoxide species can further exacerbate the degradation process, leading to the deterioration of the perovskite material.^[^ ^46^ ^]^

### 2.2 Extrinsic Degradation Pathways

External factors that cause the degradation of Sn‐containing perovskite are primarily environmental, including light, heat, moisture, oxygen, and a combination of these factors. Understanding the mechanisms behind the degradation caused by these factors is crucial for developing effective strategies to enhance the stability and performance of Sn‐containing PSCs.

#### 2.2.1 Light‐Induced Degradation

The light stability of a semiconductor photo‐absorber is crucial for its application as a solar cell. Hermetic sealing of PSCs can prevent environmental stresses, such as oxygen and moisture. Still, the absorber layer of a solar cell must possess intrinsic photostability to withstand light exposure without degradation for a lifespan of more than 25 years.^[^ ^47^ ^]^ Despite numerous studies showing the continuously improving stability of Sn‐containing PSCs with proper encapsulation or measured under the inert environment during prolonged illumination, there are still a significant amount of research reports documenting alterations to the perovskite structure induced by light exposure and degradation in device performance of Sn‐containing PSCs after prolonged light soaking.^[^ ^48^, ^49^, ^50^, ^51^, ^52^, ^53^, ^54^ ^]^ An illustrative instance is that light soaking has frequently exhibited a short‐lived constructive effect on device performance.^[^ ^50^, ^55^ ^]^ However, when subjected to extended irradiation periods, most Sn‐containing PSCs have demonstrated irreversible degradation, ascribed to the photodecomposition of Sn‐containing perovskites and the deterioration of their surfaces. Consequently, gaining a more comprehensive understanding of the light‐induced degradation mechanism is critical for advancing Sn‐containing PSCs.

**Figure 2:**
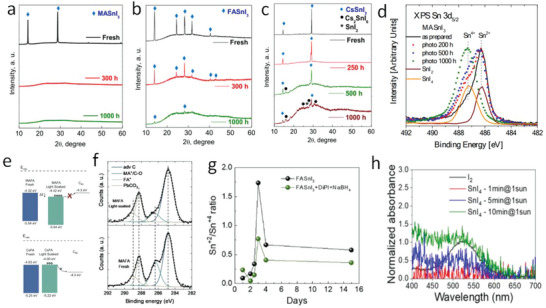
Light induced degradation of Sn‐containing perovskites. Evolution of XRD patterns of MASnI_3_ a) FASnI_3_ b) and CsSnI_3_ c) under illumination. Reproduced with permission.^[^ ^53^ ^]^Copyright 2019, American Chemical Society. d) Evolution of the Sn^2+^/Sn^4+^ ratio of Sn perovskite upon different illumination times. Reproduced with permission.^[^ ^53^ ^]^ Copyright 2019, American Chemical Society. e) Band diagram of fresh and light‐soaked MA‐containing and MA‐free Sn‐Pb perovskite films. Reproduced with permission under the terms of the CC‐BY Creative Commons Attribution‐NonCommercial 3.0 Unported license (https://creativecommons.org/licenses/by‐nc/3.0/).^[^ ^54^ ^]^ Copyright 2022, The Authors, published by Royal Society of Chemistry. f) XPS spectra of C 1s peaks in fresh and light‐soaked MA‐containing Sn‐Pb perovskite films. Reproduced with permission under the terms of the CC‐BY Creative Commons Attribution‐NonCommercial 3.0 Unported license (https://creativecommons.org/licenses/by‐nc/3.0/).^[^ ^54^ ^]^ Copyright 2022, The Authors, published by Royal Society of Chemistry. g) Evolution of the Sn^2+^/Sn^4+^ ratio of Sn perovskite upon different illumination times. Reproduced with permission.^[^ ^50^ ^]^ Copyright 2022, Elsevier. h) Normalized absorption spectra of the photogenerated I_2_ upon illumination and the reference gas phase I_2_. Reproduced with permission.^[^ ^50^ ^]^ Copyright 2022, Elsevier.

The A‐site composition is a critical factor influencing the light stability of Sn‐containing PSCs. Troshin et al.^[^ ^53^ ^]^ investigated the thermal and photodegradation of Sn‐perovskites based on various monovalent cations, including MA^+^, FA^+^, and Cs^+^. The photobleaching effects were detected in MASnI_3_ and FASnI_3_ perovskites after light soaking for ≈200 and 1 000 h, respectively, revealing the degradation of these Sn‐perovskites under prolonged illumination and the relatively better robustness of FASnI_3_ than MASnI_3_ against the light‐induced degradation. In contrast to Sn‐perovskites with organic cations, the photobleaching phenomenon was not observed in CsSnI_3_ perovskites after 1000 h of light soaking, showing much‐improved photostability in inorganic Sn‐perovskites. To gain more insight into the light‐induced degradation mechanism, X‐ray diffraction (XRD) was employed to examine the structural and compositional changes in MASnI_3_, FASnI_3_, and CsSnI_3_ perovskite films under illumination (**Figure** **2**a–c). After 300 h of aging, the XRD diffractions of the perovskite phase MASnI_3_ diminished, with no crystalline products but a broad bump observed (Figure 2a). The result shows that the MASnI_3_ perovskite underwent photodecomposition to amorphous products containing Sn^0^ and SnI_4_ as the major degradation products via Sn^2+^ disproportionation, confirmed by the X‐ray photoelectron spectroscopy (XPS) measurements (Figure 2d). The loss of MA from MASnI_3_ through volatile MA and HI/I_2_ gas species is similar to the decomposition process observed in MAPbI_3_ under illumination.^[^ ^56^, ^57^ ^]^ In contrast, FASnI_3_ and CsSnI_3_ perovskites exhibited better photostability and different degradation pathways than MASnI_3_ under illumination. Despite the formation of impurity phases, weak XRD signatures of the perovskite phase persisted in FASnI_3_ and CsSnI_3_ after 1000 h of light soaking (Figure 2b,c).

Baran et al.^[^ ^54^ ^]^ employed ultra‐violet spectroscopy (UPS) and XPS techniques to explore the photo‐induced changes in the electronic properties and chemical composition of the surface of MA‐containing (FA/MA) and MA‐free (Cs/FA) Sn–Pb perovskites. The UPS analysis showed the CBM/VBM of the FA/MA film shifted down for ≈0.1 eV after prolonged light soaking, whereas no significant changes were found in the Cs/FA film (Figure 2e). The changes affect the electronic band structure at the heterojunction, deteriorating device performance. The XPS analysis further revealed loss of MA in the FA/MA film due to the volatilization of MA from the perovskite surface (Figure 2f) but negligible changes in Sn^4+^ and I peaks in both samples upon light soaking. The results demonstrate the vulnerability of MA cations in the Sn‐containing perovskites against light is a potential cause for the loss of solar cell performance observed under illumination.

SnI_4_, a common impurity in the starting material and the decomposition product of Sn‐containing perovskites, has been found to contribute to the light‐induced degradation process. To shed light on this process, Sanchez–Diaz et al.^[^ ^50^ ^]^ studied the evolution of the Sn^2+^/Sn^4+^ ratio in FASnI_3_ perovskite samples using XPS following exposure to varying durations of light irradiation (Figure 2g). The Sn^2+^/Sn^4+^ ratio increased with the illumination and was found to be the most pronounced after three days of light soaking, indicating the decomposition of SnI_4_ into SnI_2_ and I_2_ upon exposure to light. Heavy metal complexes, such as metal halides, may exhibit metal‐to‐ligand or ligand‐to‐metal charge transfer transitions.^[^ ^50^, ^58^ ^]^ These transitions are particularly pronounced when there is a significant disparity in the intrinsic electron donating or withdrawing properties of the metal and ligand, respectively, as reflected in their respective redox potentials. The resulting charge transfer processes are often associated with optical activity and can be interpreted as intramolecular photo‐induced electronic transitions between the metallic center and the ligands or vice versa. As previously mentioned, the redox potential of the Sn^4+^/Sn^2+^ couple is +0.15 V, significantly lower than the typical chemical potential (or quasi‐Fermi energy) difference of photoexcited charge carriers under illumination. Therefore, the observed decrease in the Sn^4+^ content under illumination was attributed to the photo‐induced reduction of Sn^4+^ to Sn^2+^. This reduction resulted in the formation of I_2_, as confirmed by the detection of the I_2_ signal shown in Figure 2h. The formed I_2_ exhibits a propensity for migration to the perovskite surface.^[^ ^59^, ^60^ ^]^ Once on the surface, it may sublimate or contribute to the further degradation of Sn‐containing perovskites.^[^ ^43^ ^]^

#### 2.2.2 Heat‐Induced Degradation

Solar modules under direct sunlight outdoors typically have an average operating temperature of ≈50 °C.^[^ ^61^ ^]^ Modules on the field may be heated to a high temperature of 85 °C in summer, depending on environmental conditions.^[^ ^62^ ^]^ Therefore, robust thermal stability is essential to ensure the durability of a solar cell under real‐world conditions. Commercial solar modules have to successfully undergo the temperature cycling and damp heat tests required by the International Electrotechnical Commission's (IEC 61 215) specifications before entering the market.^[^ ^63^ ^]^

The thermal stability requirements present a great challenge to PSCs, particularly those based on Sn‐containing perovskites, because they exhibit much inferior thermal stability compared with their Pb‐based counterparts and other conventional inorganic semiconductors. To meet the thermal stability criteria of solar cells, it is crucial to understand the degradation mechanism of Sn‐containing perovskites under thermal stress.

**Figure 3:**
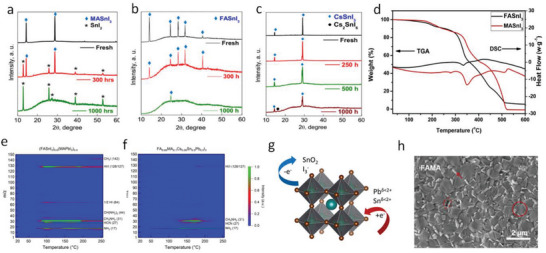
Heat induced degradation of Sn‐containing perovskites. Evolution of XRD patterns of MASnI_3_ a) FASnI_3_ b) and CsSnI_3_ c) under thermal annealing at ≈90 °C. Reproduced with permission.^[^ ^53^ ^]^ Copyright 2019, American Chemical Society. d) TGA and DSC spectra of Sn perovskites. Reproduced with permission.^[^ ^67^ ^]^ Copyright 2016, Wiley‐VCH. Temperature‐programmed desorption mass spectrometry of (FASnI_3_)_0.6_(MAPbI_3_)_0.4_ e) and FA_0.85_MA_0.1_Cs_0.05_Sn_0.5_Pb_0.5_I_3_ f) perovskite films. Reproduced with permission.^[^ ^51^ ^]^ Copyright 2020, Nature Publishing Group. g) Schematic of proposed chemical degradation mechanism of Sn–Pb perovskite. Reproduced with permission.^[^ ^45^ ^]^ Copyright 2020, American Chemical Society. h) The SEM image of a Sn‐Pb perovskite film after thermal stressing at 85 °C. Reproduced with permission.^[^ ^68^ ^]^ Copyright 2022, Wiley‐VCH.

Similar to their Pb counterparts,^[^ ^64^, ^65^, ^66^ ^]^ Sn‐containing perovskite absorber layers undergo thermal decomposition when subjected to heat. Using XRD, Troshin et al.^[^ ^53^ ^]^ investigated the thermal degradation of MASnI_3_, FASnI_3_, and CsSnI_3_ perovskite films at ≈90 °C for 1000 h (**Figure** **3**a–c). The thermal aging of the MASnI_3_ film resulted in the formation of the perovskite decomposition products, SnI_2_ and MAI, as well as unidentified amorphous phases, likely related to SnI_4_ (Figure 3a). In contrast, the FASnI_3_ film under thermal stress exhibited no SnI_2_ peaks but a gradual decay of the perovskite peaks and the appearance of amorphous products (Figure 3b). The CsSnI_3_ film showed the best thermal stability among the group, retaining the primary perovskite phase with minor Cs_2_SnI_6_ and amorphous phases appealing after 1000 h of thermal stress (Figure 3c). The poor thermal stability of MASnI_3_ and FASnI_3_ is likely associated with their polycrystalline features with a high surface area and a high density of grain boundaries. Dang et al.^[^ ^67^ ^]^ carried out thermogravimetric analysis (TGA) and differential scanning calorimetry (DSC) measurements on MASnI_3_ and FASnI_3_ single crystals (Figure 3d). Surprisingly, they found that the decomposition temperature of MASnI_3_ at 200 °C is higher than that of FASnI_3_ at 175 °C. It is worth noting that the decomposition temperatures of Sn‐perovskite single crystals are significantly higher than that of their polycrystalline thin films. The results reveal that the Sn‐containing perovskite single crystals could be intrinsically stable under typical solar cell operation temperatures, and thermal instability mainly originates from grain boundaries and interfaces.

Temperature‐programmed desorption mass spectrometry (TPD‐MS) technique was employed to probe the volatile products of the thermal decomposition of Sn‐containing perovskites.^[^ ^51^ ^]^ The TPD‐MS analysis of (FASnI_3_)_0.6_(MAPbI_3_)_0.4_ films revealed the release of methylamine (CH_3_NH_2_) and HI gas species at a low temperature of ≈68 °C (Figure 3e). In contrast, an FA_0.85_MA_0.1_Cs_0.05_Sn_0.5_Pb_0.5_I_3_ perovskite film with reduced MA and less Sn exhibited much improved thermal stability with a higher thermal decomposition temperature at ≈125 °C (Figure 3f). The results reveal the importance of tailoring the Sn‐containing perovskite composition to enhance its thermal stability.

A detailed chemothermal study of the Sn‐Pb perovskites under thermal treatment in ambient and inert environments was reported by Ratcliff et al.^[^ ^45^ ^]^ The work proposed surface‐activated corrosion in Sn‐Pb perovskites involving the formation I_3_^−^ intermediates prior to I_2_ escape and under‐coordinated metal sites, i.e., Sn^δ < 2+^ and Pb^δ < 2+^, on the surface (Figure 3g). The corrosion processes on the surface and at grain boundaries were mainly attributed to the chemical equilibrium among different oxidation states of Pb and Sn as well as the redox complexity of various I species. The Sn‐Pb reaction and formation of $I_{3}^{-}$ were observed during the thermal annealing, resulting from the following reaction equations:

(8) $${\mathrm{Pb}}^{2+}+{\mathrm{Sn}}^{2+}⇄{\mathrm{Pb}}^{0}+{\mathrm{Sn}}^{4+}$$

(9) $$2I^{-}⇄I_{2}+2e^{-}$$

(10) $$I^{-}+I_{2}⇄I_{3}^{-}$$

As mentioned above, thermal degradation of the Sn‐containing perovskite can release decomposition gas products and cause corrosion on the surface, which may cause the formation of voids, secondary phase, and cleavage on the surface of perovskite (Figure 3h).^[^ ^68^ ^]^

#### 2.2.3 Moisture‐Induced Degradation

**Figure 4:**
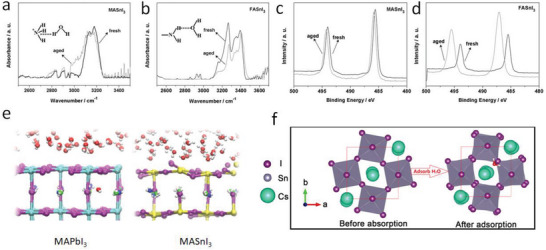
Water induced degradation of Sn‐containing perovskites. FTIR spectra of fresh and aged MASnI_3_ a) and FASnI_3_ b) perovskite films subjected to air exposure. Reproduced with permission.^[^ ^69^ ^]^ Copyright 2016, Wiley‐VCH. XPS Sn3d peaks of fresh and aged MASnI_3_ c) and FASnI_3_ d) perovskite films subjected to air exposure. Reproduced with permission.^[^ ^69^ ^]^ Copyright 2016, Wiley‐VCH. e) Time‐averaged structure of MAPbI_3_/water and MASnI_3_/water interface. Reproduced under the terms of the CC‐BY Creative Commons Attribution 4.0 International license (https://creativecommons.org/licenses/by/4.0/).^[^ ^71^ ^]^ Copyright 2022, The Authors, published by American Chemical Society. f) Schematic illustration showing changes in the geometrical structure in CsSnI_3_ perovskites after absorption of water. Reproduced with permission.^[^ ^72^ ^]^ Copyright 2020, Japan Society of Applied Physics.

Due to their hygroscopic properties, Sn‐containing perovskites are sensitive to moisture. Zhao et al.^[^ ^69^ ^]^ utilized Fourier transform infrared (FTIR) spectroscopy to investigate the interaction between H_2_O and organic cations in MASnI_3_ and FASnI_3_ perovskites. The MASnI_3_ perovskite showed a new broad shoulder at ≈3000 cm^−1^ after exposure to moisture (**Figure** **4**a). This observed spectral signature bears a strong resemblance to the characteristic signatures of NH_4_^+^, indicating that the interaction between MASnI_3_ perovskite and the H_2_O molecule may occur through hydrogen bonding between the N atom in the MA^+^ cation and the H atom in the H_2_O molecule. In contrast to MASnI_3_, the FASnI_3_ showed the reduced stretching vibration of –NH_2_, indicating an interaction between H_2_O molecules and FASnI_3_ perovskite via hydrogen bonding between the H atom in NH_2_ and the O atom in H_2_O (Figure 4b). It is important to note that the interaction of H_2_O molecules and ammonium groups in Sn‐containing perovskites can create vacancies for oxygen ingress and iodine egress, providing the pathway for further degradation.

Aside from reacting with organic halides, H_2_O can also impact the Sn‐I bond in Sn‐containing perovskites. An XPS analysis showed that the Sn‐3d_5/2_ peak of MASnI_3_ and FASnI_3_ films shifted by 0.4 and 1.6 eV towards higher binding energy, respectively, after exposure to water (Figure 4c,d), indicating the Sn^2+^ to Sn^4+^ transitions.^[^ ^69^ ^]^ It is worth noting that the large energy shift in FASnI_3_ could be due to the stronger coordination with a stronger electronegative atom, such as Sn–O, than Sn–I. Hu et al. conducted density functional theory (DFT) calculations and showed that H_2_O molecules prefer to adsorb on the surface of MASnI_3_ via a hydrogen bond with I, weakening the Sn–I bond on the surface.^[^ ^70^ ^]^ De Angelis et al.^[^ ^71^ ^]^ utilized ab initio molecular dynamics simulations to visualize the water interaction with MAPbI_3_ and MASnI_3_. They found that MAPbI_3_ was only weakly distorted by water, whereas the MASnI_3_ surface showed a strong structural distortion with the SnI_2_‐terminated MASnI_3_ surface readily dissolved by water (Figure 4e). In this process, water molecules form bonds with the Sn atoms on the surface, breaking the axial Sn–I bond with the I atoms in the neighboring MAI layer. All‐inorganic CsSnI_3_ is also vulnerable to water‐induced degradation. Yang et al.^[^ ^72^ ^]^ performed DFT calculations and found out that water adsorbed on the surface of γ‐phase CsSnI_3_ could induce charge transfers between the H atom in the water and adjacent I atoms, and between the O atom and Cs+, leading to distortion of SnI_6_ octahedra and structural deformation (Figure 4f).

#### 2.2.4 Oxygen‐Induced Degradation

**Figure 5:**
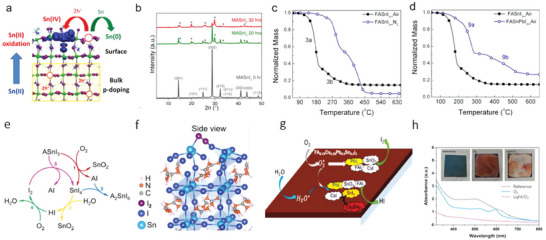
Oxygen and multi‐factor induced degradation of Sn‐containing perovskites. a) Schematic illustration of defects formation for the Sn perovskite upon oxidation. Reproduced with permission.^[^ ^73^ ^]^ Copyright 2020, American Chemical Society. b) Evolution of XRD patterns of MASnI_3_ perovskite films in dry air. Reproduced with permission.^[^ ^74^ ^]^ Copyright 2021, American Chemical Society. c) Thermogravimetric analysis of FASnI_3_ powders in N_2_ and air. Reproduced with permission.^[^ ^75^ ^]^ Copyright 2017, American Chemical Society. d) Thermogravimetric analysis of FASnI_3_ and FASn_0.5_Pb_0.5_I_3_ powders in air. Reproduced with permission.^[^ ^75^ ^]^ Copyright 2017, American Chemical Society. e) Schematic illustration of degradation mechanism of the Sn perovskite under ambient air exposure. Reproduced under the terms of the CC‐BY Creative Commons Attribution 4.0 International license (https://creativecommons.org/licenses/by/4.0/).^[^ ^33^ ^]^ Copyright 2021, The Authors, published by Nature Publishing Group. f) The side view of the structural arrangement of an absorbed I_2_ molecule on the (001) surface of the FASnI_3_ perovskite. Reproduced under the terms of the CC‐BY Creative Commons Attribution 4.0 International license (https://creativecommons.org/licenses/by/4.0/).^[^ ^33^ ^]^ Copyright 2021, The Authors, published by Nature Publishing Group. g) Schematic illustration of degradation mechanism of the Sn‐Pb perovskite under ambient air exposure. Reproduced with permission.^[^ ^76^ ^]^ Copyright 2023, American Chemical Society. h) UV‐vis absorption spectra of the reference FASnI_3_ film and FASnI_3_ films with O_2_ and light/O_2_ exposure. Reproduced with permission.^[^ ^46^ ^]^ Copyright 2022, Royal Society of Chemistry.

The stability of Sn‐containing perovskites is considerably impacted by oxygen due to the propensity of Sn^2+^ to undergo oxidation towards the more stable Sn^4+^ state. This oxidation of Sn^2+^ is anticipated to be highly unfavorable within the bulk of Sn‐containing perovskites, while it is energetically favored at perovskite surfaces (**Figure** **5**a).^[^ ^73^ ^]^ As a result, Sn^4+^ in bulk undergoes spontaneous transformation to Sn^2+^, releasing two holes to the valence band and p‐doping of the perovskite. Meanwhile, the presence of Sn^4+^ on the surface can facilitate the degradation of the lattice to secondary phases, such as vacancy‐ordered double perovskites (Figure 5b).^[^ ^74^ ^]^

To study the degradation pathway of Sn‐containing perovskite upon exposure to oxygen, McGehee et al.^[^ ^75^ ^]^ utilized TGA to investigate the oxidative products (Figure 5c,d). The study revealed that the decomposition products of Sn perovskites consisted of organic halides, SnI_4_ and SnO_2_. However, the decomposition products of Sn–Pb perovskites included organic halides, SnO_2,_ and I_2_, indicating a distinct degradation pathway for Sn–Pb perovskites compared to the pure Sn perovskites. This is likely related to the chemical equilibrium among different oxidation states of Pb and Sn, as discussed above.

#### 2.2.5 Multi‐Factors‐Induced Degradation

The environmental stresses discussed above are all isolated. However, in the real‐world scenario, solar cells are exposed to combinations of these stresses, which can lead to accelerated degradation or even different degradation mechanisms. Therefore, comprehending the mechanisms of degradation induced by multiple factors is crucial for the commercialization of PSCs.

Haque et al.^[^ ^33^ ^]^ conducted a comprehensive study and proposed a cyclic degradation mechanism of Sn perovskite under ambient air exposure (Figure 5e). The first step of Sn perovskite degradation in ambient air is consistent with the results of oxygen‐induced degradation discussed in the previous section, as described by reaction (11):

(11) $$2{\mathrm{ASnI}}_{3}+O_{2}\to {\mathrm{SnI}}_{4}+{\mathrm{SnO}}_{2}+2\mathrm{AI}$$

where A represents the organic monovalent cations. The presence of SnI_4_ is highly detrimental to the optoelectronic properties and stability of the perovskite films. The accumulation of SnI_4_ and AI then triggers the second step of degradation towards the formation of vacancy‐ordered double perovskites, as shown in reaction (12):

(12) $${\mathrm{SnI}}_{4}+2\mathrm{AI}\to A_{2}{\mathrm{SnI}}_{6}$$

Alternatively, the SnI_4_ can spontaneously react with water to form HI gas and solid residue SnO_2_, as detailed in reaction (13):

(13) $${\mathrm{SnI}}_{4}+2H_{2}O\to 4\mathrm{HI}+{\mathrm{SnO}}_{2}$$

The HI gas further reacts with oxygen, as shown in reaction (14):

(14) $$4\mathrm{HI}+O_{2}\to 2I_{2}+2H_{2}O$$

Iodine generated in reaction (14) can adsorb on the surface of perovskite, break the Sn‐I bonds, and deteriorate the surface of Sn‐perovskite (Figure 5f), which further oxidizes the Sn perovskite, as detailed in reaction (15):

(15) $${\mathrm{ASnI}}_{3}+I_{2}\to {\mathrm{SnI}}_{4}+\mathrm{AI}$$

This reaction is energetically favorable with a formation energy of −0.61 eV. More importantly, the degradation product SnI_4_ can participate again in the reaction (13), leading to a cyclic degradation mechanism.

A similar work was reported by Hillhouse et al.,^[^ ^76^ ^]^ revealing the degradation mechanism of FA/Cs‐based Sn‐Pb perovskites under oxygen and moisture exposure (Figure 5g). The dry oxidation pathway leads to the decomposition products of PbI_2_, SnO_2_, FAI, CsI, and I_2_, whereas the water‐accelerated oxidation pathway produces additional decomposition products, such as SnI_4_, Cs_2_SnI_6_, and HI.

Zhao et al.^[^ ^46^ ^]^ observed more severe degradation of Sn perovskite under exposure to light and oxygen than oxygen only (Figure 5h). After a comprehensive study, the authors attributed the accelerated degradation to superoxide generated by photoexcitation at the iodine vacancy sites with the assistance of water. This report manifests the importance of suppressing superoxide formation to enhance the durability of Sn‐perovskites.

### 2.3 Interface and Contact Materials

The charge transport layer that forms an interface with the perovskite absorber layer is a potential instability origin that remains active throughout the lifespan of Sn‐containing PSCs. In the normal (n‐i‐p) structure of Sn‐containing PSCs, metal oxides, e.g., titanium dioxide (TiO_2_), are commonly used as the electron transport layer (ETL). It has been observed that TiO_2_ tends to generate electron‐hole pairs easily under UV light illumination. The resulting electrons in the conduction band of TiO_2_ tend to react with surface‐bound superoxide, leading to the desorption of oxygen molecules and creating oxygen vacancies. Consequently, this process induces the oxidation of Sn^2+^ to Sn^4+^ at the interface of Sn‐perovskites.^[^ ^77^, ^78^ ^]^ Regarding the hole transport layer (HTL), 2,2′,7,7′‐Tetrakis[N,N‐di(4‐methoxyphenyl)amino]−9,9′‐spirobifluorene (Spiro‐OMeTAD) has been extensively employed as the primary HTL in n‐i‐p structure due to its decent hole mobility and band alignment with Pb‐perovskites. However, the conductivity of Spiro‐OMeTAD relies on its oxidation, which necessitates an oxidation process to enhance its charge transport capabilities. In addition, the presence of the lithium salt, serving as an initiator for Spiro‐OMeTAD oxidation, also accelerates the oxidation of Sn^2+^ at the interface.^[^ ^79^ ^]^

In the inverted (p‐i‐n) structure of Sn‐containing PSCs, the widely used hole transport layer, Poly(3,4‐ethylenedioxythiophene):poly(styrenesulfonate) (PEDOT:PSS), has been found to adversely affect device stability. Huang et al.^[^ ^43^ ^]^ conducted a study where they observed the formation of iodine in the Sn‐Pb film deposited on the PEDOT:PSS substrate when subjected to an elevated temperature at 85 °C for 2 days. In contrast, significantly lower levels of iodine signal were detected in the Sn‐Pb film deposited directly on the bare indium tin oxide (ITO) substrate. This observation suggests that the acidic nature of PEDOT:PSS can accelerate the oxidation of iodide anions present at the buried interface, leading to the formation of iodine. Consequently, the generated iodine species can further promote the oxidation of Sn^2+^ to Sn^4+^ in the perovskite layer.

Silver (Ag) is commonly employed as the back electrode in Sn‐containing PSCs due to its favorable characteristics, such as low work function, high reflectivity across the visible and near‐infrared spectrum, and low electrical resistivity. However, several studies have indicated that the Ag electrode can contribute to device instability.^[^ ^80^, ^81^, ^82^ ^]^ For example, Hatton et al.^[^ ^82^ ^]^ investigated the impact of the Ag electrode on the stability of Sn PSCs under ambient air and 1 sun illumination conditions. It was observed that the metallic Ag color of the electrode turned brown after testing in ambient air for 14 h, indicating corrosion of the Ag electrode. This corrosion of the Ag electrode was attributed to its reaction with I_2_ gas, which is produced as a result of the decomposition of SnI_4_ in the presence of H_2_O or light, as mentioned earlier.

## 3 Strategies for Improving the Stability of Sn‐Containing PSCs

Over the last few years, considerable effort has been dedicated to mitigating the degradation and enhancing the stability of Sn‐containing PSCs, yielding significant advancements in device durability. A survey of recent publications on advanced strategies for stabilizing Sn‐containing PSCs and their impact on improving device stability are summarized in **Table** **1**. This section focuses on an overview and analysis of several advanced strategies employed to enhance the stability of Sn‐containing PSCs, including antioxidant additives and reducing agents, compositional engineering, surface passivation, device architecture innovations, and encapsulation engineering.

**Table 1:** Summary of strategies to improve stability of Sn‐containing PSCs.

Sn Perovskite	Strategy	Stability condition	Stability	Retention	Ref.
FASnI_3_	Antioxidant (KHQSA)	Unencapsulated in ambient air (20% RH)	500 h	80%	[85]
FASnI_3_	Antioxidant (5‐AVAI)	Encapsulated at MPP in ambient air (50% RH)	100 h	100%	[130]
FASnI_3_	Antioxidant (PVA)	Encapsulated at close to MPP in ambient air, with a UV filter	400 h	100%	[131]
FASnI_3_	Antioxidant (GA)	Unencapsulated in ambient air (20% RH)	1000 h	80%	[132]
FASnI_3_	Antioxidant (2‐F‐PEAI)	Unencapsulated at continuous illumination in N_2_	1600 h	85%	[133]
FASnI_3_	Antioxidant (FOEI)	Encapsulated at MPP in ambient air, with a UV filter	500 h	100%	[134]
FASnI_3_	Antioxidant (FBH)	Encapsulated at MPP in ambient air (20% RH)	600 h	93%	[135]
Cs_0.2_FA_0.8_SnI_3_	Antioxidant (SnF_2_, SnCl_2_)	Encapsulated at MPP	1000 h	95%	[49]
PEA_0.15_FA_0.85_SnI_3_	Antioxidant (GAA)	Shelf‐stability in N_2_	1200 h	93%	[136]
FA_0.75_MA_0.25_SnI_2.75_ Br_0.25_	Antioxidant (4A3HA)	Thermal stability at 85 °C in N_2_	1000 h	80%	[137]
FASnI_3_	Reducing agent (PHCl)	Shelf‐stability in N_2_	110 d	100%	[138]
FA_0.75_MA_0.25_SnI_3_	Reducing agent (TM‐DHP)	Shelf‐stability in N_2_	50 d	100%	[91]
FASnI_3_	Reducing agent (DipI+NaBH_4_)	Unencapsulated at MPP in N_2_	1300 h	96%	[50]
CsSnI_3‐_ *_x_* Br*_x_*	Reducing agent (DMKO)	Shelf‐stability in N_2_	1000 h	80%	[139]
FASnI_3_	A‐site composition (Reduced MA^+^)	Encapsulated at MPP in ambient air	1000 h	83%	[48]
GA*_x_* FA_1‐_ *_x_*_‐2_ *_y_* SnI_3_‐yEDAI2	A‐site composition (GA^+^, EDA^2+^)	Shelf‐stability in N_2_	2000 h	131.50%	[140]
PPA*_x_* FA_1‐_ *_x_* SnI_3_	A‐site composition (PPA^+^)	Shelf‐stability in N_2_	1440 h	92%	[141]
FASnI_3_	A‐site composition (FPEA^+^)	Shelf‐stability in N_2_	432 h	80%	[99]
PEA*_X_* FA_0.75_MA_0.25‐_ *_X_* SnI_2_Br	A‐site composition (PEA^+^)	Shelf‐stability in ambient air (25‐30% RH)	300 h	80%	[96]
PEA_0.15_FA_0.85_SnI_3_	Halide engineering (NH_4_SCN)	Shelf‐stability in N_2_	600 h	90%	[101]
FASnI_3_	Interface passivation (PEABr)	Encapsulated at MPP in ambient air	350 h	80%	[104]
GA_0.2_FA_0.8_SnI_3_	Interface passivation (AN)	Shelf‐stability in air (Without encapsulation)	150 h	100%	[105]
FASnI_3_	Interface passivation (PAI)	Unencapsulated at MPP in N_2_	1000 h	95%	[106]
FA_0.75_MA_0.25_SnI_3_	Interface passivation (FACl)	Shelf‐stability in N_2_	1000 h	92%	[107]
FASnI_3_	Interface passivation (MHATFA)	Unencapsulated at MPP in N_2_	1000 h	76.4%	[108]
CsSnI_3_	Interface passivation (TSC)	Encapsulated at MPP in air	500 h	90%	[110]
{en}FASnI_3_	Device architecture innovations (BDT)	Shelf‐stability in N_2_	14 m	66.9%	[116]
FASnI_3_	Device architecture innovations (HTL‐free)	MPP in N_2_	40 d	95%	[118]
FASnI_3_	Device architecture innovations (HTL‐free)	Thermal stability at 80 °C in N_2_	300 h	90%	[118]
FASnI_3_	Device architecture innovations (PPr‐SBT‐14)	Shelf‐stability in N_2_	6000 h	100%	[120]
FASnI_3_	Device architecture innovations (C_60_‐BPy)	Unencapsulated at MPP in N_2_	1000 h	95%	[122]
FA_0.5_MA_0.5_Sn_0.5_ Pb_0.5_I_3_	Antioxidant (AA)	Shelf‐stability in N_2_	1 month	99%	[142]
FA_0.7_MA_0.3_Sn_0.5_ Pb_0.5_I_3_	Antioxidant (FSA)	Shelf‐stability in ambient air (<20% RH)	500 h	95%	[86]
Cs_0.3_FA_0.7_Sn_0.3_Pb_0.7_I_3_	Antioxidant (SnCl_2_·3FACl)	Unencapsulated at MPP in N_2_	750 h	80%	[143]
Cs_0.05_FA_0.7_MA_0.25_ Sn_0.5_Pb_0.5_I_3_	Antioxidant (TMSI)	Unencapsulated at MPP in N_2_	1200 h	88%	[87]
FA_0.7_MA_0.3_Sn_0.5_ Pb_0.5_I_3_	Reducing agent (Sn powder)	Unencapsulated tandem cells at MPP in N_2_	463 h	90%	[89]
FASn_81.3_Pb_18.7_I_3_	Reducing agent (Pb powder)	Unencapsulated at MPP in N_2_	700 h	81%	[90]
EA_0.05_FA_0.5_MA_0.45_Sn_0.5_Pb_0.5_I_3_	Reducing agent (HBA)	Unencapsulated at light‐soaking condition in N_2_	150 h	90%	[26]
Cs_0.2_FA_0.8_Sn_0.5_Pb_0.5_I_3_	Reducing agent (BHC)	Encapsulated at continuous illumination in ambient air (30‐65% RH)	250 h	94%	[93]
Cs_0.05_FA_0.85_MA_0.1_ Sn_0.5_Pb_0.5_I_3_	A‐site composition (Reduced MA^+^)	Encapsulated at MPP in ambient air	450 h	92%	[51]
Cs_0.05_FA_0.85_MA_0.1_ Sn_0.5_Pb_0.5_I_3_	A‐site composition (Reduced MA^+^)	Thermal stability at 85 °C in N_2_	360 h	70%	[51]
Cs_0.025_FA_0.475_MA_0.5_ Sn_0.5_Pb_0.5_I_2.925_ Br_0.075_	A‐site composition (Rb^+^)	Thermal stability at 85 °C in N_2_	150 h	75%	[144]
Cs_0.17_FA_0.83_Sn_0.5_ Pb_0.5_I_3_	A‐site composition (Cs^+^)	Unencapsulated at MPP in N_2_	100 h	82%	[145]
Cs_0.25_FA_0.75_Sn_0.5_ Pb_0.5_I_3_	A‐site composition (Cs^+^)	Thermal stability at 85 °C in N_2_	500 h	95.1%	[94]
CsSn_0.6_Pb_0.4_I_2.4_ Br_0.6_	A‐site composition (Cs^+^)	Thermal stability at 85 °C in N_2_	700 h	72%	[95]
FA_0.6_MA_0.4_Sn_0.6_ Pb_0.4_I_3_	A‐site composition (PEA^+^, GA^+^)	Unencapsulated at continuous illumination in N_2_	1830 h	82%	[52]
Cs_0.2_FA_0.8_Sn_0.5_Pb_0.5_I_3_	Halide engineering (OABF_4_)	Thermal stability at 85 °C in N_2_	720 h	80%	[102]
Cs_0.2_FA_0.8_Sn_0.5_Pb_0.5_I_3_	Halide engineering (OABF_4_)	Encapsulated at MPP in ambient air	1000 h	88%	[102]
Cs_0.1_FA_0.6_MA_0.3_ Sn_0.5_Pb_0.5_I_3_	Interface passivation (PP, CPTA)	Shelf‐stability in N_2_	2000 h	96%	[111]
FASn_0.5_Pb_0.5_I_3_	Interface passivation (OH‐PEABr)	Unencapsulated at MPP in N_2_	800 h	80%	[113]
Cs_0.05_FA_0.7_MA_0.25_ Sn_0.5_Pb_0.5_I_3_	Interface passivation (PC)	Encapsulated at MPP in N_2_	400 h	80%	[114]
Cs_0.1_FA_0.6_MA_0.3_ Sn_0.5_Pb_0.5_I_3_	Interface passivation (EDAI_2_, GlyHCl)	Unencapsulated at MPP in N_2_	200 h	80%	[115]
Cs_0.25_FA_0.75_Sn_0.5_ Pb_0.5_I_3_	Device architecture innovations (PTAA)	Thermal stability at 85 °C in N_2_	4000 h	80%	[117]
Cs_0.2_FA_0.8_Sn_0.5_ Pb_0.5_I_3_	Device architecture innovations (SnOCl)	Continuous 1‐sun illumination	1200 h	87%	[119]
Cs_0.2_FA_0.8_Sn_0.5_ Pb_0.5_I_3_	Device architecture innovations (SnOCl)	Thermal stability at 85 °C	1500 h	85%	[119]
Cs_0.25_FA_0.75_Sn_0.4_ Pb_0.6_I_3_	Device architecture innovations (HTL‐free, IZO)	Thermal stability at 85 °C in air (Without encapsulation)	1000 h	95%	[123]

### 3.1 Antioxidant and Reducing Agents

One primary factor causing the instability of Sn‐containing perovskite is the oxidation of Sn^2+^ induced by an extrinsic oxidizer, like O_2_, and an intrinsic factor, like I^0^. The oxidation of Sn^2+^ may originate from various steps of device fabrication, including source material (e.g., SnI_2_), precursor solution (e.g., DMSO), deposition process (e.g., processing environment), post‐annealing, device encapsulation, and measurement. Therefore, it is critical to incorporate antioxidant additives and reducing agents that can suppress the formation of Sn^4+^ defects in the perovskite precursors and films.

**Figure 6:**
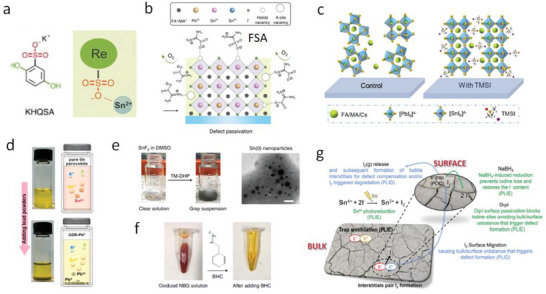
Antioxidant and reducing agents to improve stability. a) Molecular structure of KHQSA and schematic diagram showing the interaction between KHQSA and Sn^2+^ ion. Reproduced with permission.^[^ ^85^ ^]^ Copyright 2019, Wiley‐VCH. b) Schematic illustration of antioxidation at surface and grain boundary of Sn–Pb perovskite enabled by FSA. Reproduced with permission.^[^ ^86^ ^]^ Copyright 2020, Nature Publishing Group. c) Schematic diagram of Sn‐Pb perovskite with and without TMSI. Reproduced with permission.^[^ ^87^ ^]^ Copyright 2023, American Chemical Society. d) The image and schematic illustration of Sn perovskite precursor with and without Pb powder. Reproduced with permission.^[^ ^90^ ^]^ Copyright 2021, Elsevier. e) Images of the SnF_2_ solution before (left) and after (middle) incorporation of TM‐DHP and TEM image (right) of the formed Sn(0) nanoparticles. Reproduced under the terms of the CC‐BY Creative Commons Attribution 4.0 International license (https://creativecommons.org/licenses/by/4.0/).^[^ ^91^ ^]^ Copyright 2020, The Authors, published by Nature Publishing Group. f) Image of oxidized Sn‐Pb perovskite solution and reduced solution by BHC. Reproduced with permission.^[^ ^93^ ^]^ Copyright 2022, Nature Publishing Group. g) Illustration of Sn perovskite reactivity under light‐soaking condition. Reproduced with permission.^[^ ^50^ ^]^ Copyright 2022, Elsevier.

One potential approach to mitigate this Sn^2+^ oxidation issue in Sn‐containing perovskites is incorporating antioxidants. SnF_2_ is a widely used antioxidant additive in Sn‐containing perovskites, and its effect has been systematically studied.^[^ ^40^, ^83^, ^84^ ^]^ Along with SnF_2_, various molecular antioxidants have also been added to the perovskite precursor to prevent Sn^2+^ oxidation (Table 1). For instance, Yan et al.^[^ ^85^ ^]^ reported the incorporation of the potassium salt of hydroquinone sulfonic acid (KHQSA) as a benchmark antioxidant into FASnI_3_ perovskite. The KHQSA molecule contains a reducing hydroxybenzene function group to prevent Sn^2+^ oxidation and a sulfonate group (SO_3_^−^) that can coordinate with Sn^2+^ (**Figure** **6**a). Similarly, Tan et al.^[^ ^86^ ^]^ incorporated a surface‐anchoring zwitterionic antioxidant, formamidine sulfinic acid (FSA), into the Sn‐Pb perovskite precursor (Figure 6b). Owing to the reducing activity of the formamidine group (CH_4_N_2_^+^) and the chemical interaction between the sulfinic group in FSA and Sn^2+^, the oxidation of Sn^2+^ was effectively suppressed. Combined with a wide bandgap PSC, an unencapsulated all‐perovskite tandem device with FSA antioxidants retained over 95% of its original efficiency following storage in the air over 500 h. In another study, Zhou et al.^[^ ^87^ ^]^ utilized trimethylsulfoxonium iodide (TMSI) as an additive in the context of Sn‐Pb perovskites (Figure 6c). TMSI was chosen due to its unique chemical properties, specifically, its sulfoxide group that facilitates coordination with Sn^2+^ ions. This coordination interaction effectively hinders the undesired oxidation of Sn^2+^ to Sn^4+^ species. Consequently, when an unencapsulated device incorporating TMSI was subjected to ambient air storage over 200 h, 93% of its original PCE was retained.

As aforementioned, the presence of SnI_4_ in the starting materials of Sn‐containing perovskites can also contribute to the stability issue of Sn‐containing PSCs. Sn(0) powders have been added to the Sn and Sn/Pb perovskite precursor solution to purify the source materials.^[^ ^88^, ^89^ ^]^ Similarly, Fang et al.^[^ ^90^ ^]^ proposed a strategy to address this problem by introducing a reducing agent, lead powder (Pb^0^), to effectively reduce the Sn^4+^ content in the perovskite precursor (Figure 6d). Based on standard redox potentials, Pb^0^ is energetically more favorable to react with Sn^4+^ instead of Sn^2+^, resulting in a complete reduction of Sn^4+^ content in the perovskite precursor. By implementing this approach, a device achieved a PCE of 20.01% and maintained 81% of its initial efficiency after continuous operation at maximum power point (MPP) in N_2_ for 700 h, significantly superior to the device without Pb^0^ treatment. To prepare reducing agents in Sn‐perovskite precursor, Wakamiya et al.^[^ ^91^ ^]^ reacted 1,4‐Bis(trimethylsilyl)−2,3,5,6‐tetramethyl‐1,4‐dihydropyrazine (TM‐DHP) with SnF_2_ to form

Sn(0) nanoparticles (Figure 6e), which were used to scavenge for Sn^4+^ impurities in Sn‐perovskite films.

Additionally, organic reducing agents have been widely used as co‐additives in Sn‐containing perovskites. Hydrazine and its derivatives, which are highly effective reducing agents, can be used to create a reducing vapor atmosphere and incorporated into the perovskite precursor as reducing additives.^[^ ^92^ ^]^ For instance, Huang et al.^[^ ^93^ ^]^ conducted a study demonstrating the ability of benzylhydrazine hydrochloride (BHC) to facilitate the reduction of Sn^4+^ to Sn^2+^ in oxidized Sn‐Pb perovskite precursor. Incorporating BHC restored the perovskite's yellow color, indicating its successful reduction capability (Figure 6f). This reduction process effectively suppressed the formation of Sn^4+^ within the perovskite material. Consequently, the cell exhibited significantly enhanced stability, retaining 93.7% of its initial PCE even after prolonged exposure to 1 sun illumination for ≈250 h.

The formation of I_2_, a degradation product induced by light and heat, can further oxidize Sn^2+^ to Sn^4+^ in Sn‐containing perovskites. To prevent this oxidation process, Mora‐Seró et al.^[^ ^50^ ^]^ incorporated a well‐known reductant, sodium borohydride (NaBH_4_), into Sn perovskite films (Figure 6g). NaBH_4_ can effectively suppress the formation of I_2_ in the Sn‐perovskite films when exposed to illumination through a reducing reaction as follows:

(16) $${\mathrm{NaBH}}_{4}+I_{2}\to \mathrm{NaI}+{\mathrm{BH}}_{3\left(g\right)}+{\mathrm{HI}}_{\left(g\right)}$$

Benefiting from the reduced I_2_ formation, the device with NaBH_4_ additive exhibited superior light stability under MPP tracking, maintaining 95% of its initial efficiency for more than 1300 h.

### 3.2 Compositional Engineering

#### 3.2.1 Reducing MA Content

**Figure 7:**
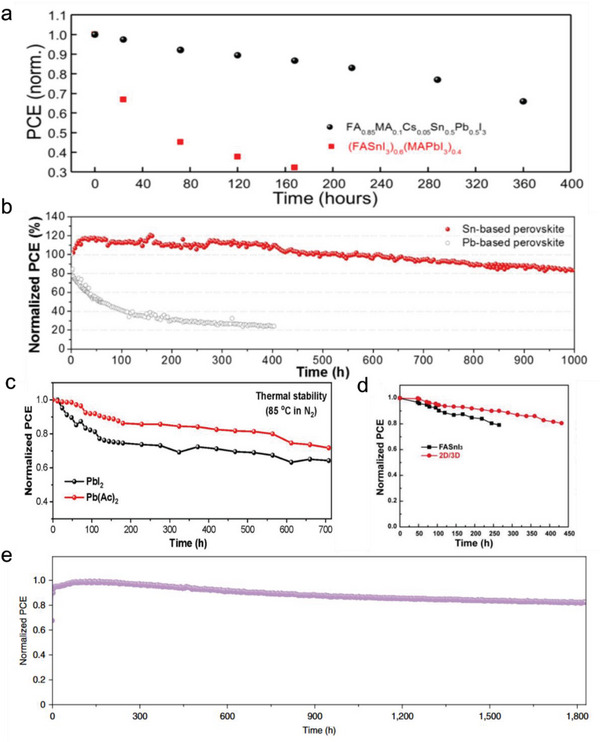
A‐site composition engineering to improve stability. a) Thermal stability of (FASnI_3_)_0.6_(MAPbI_3_)_0.4_ and FA_0.85_MA_0.1_Cs_0.05_Sn_0.5_Pb_0.5_I_3_ PSCs aged at 85 °C. Reproduced with permission.^[^ ^51^ ^]^ Copyright 2020, Nature Publishing Group. b) Long‐term light stability of Sn‐based and Pb‐based PSCs under continuous light illumination. Reproduced with permission.^[^ ^48^ ^]^ Copyright 2018, American Chemical Society. c) Thermal stability of CsPb_0.4_Sn_0.6_I_2.4_Br_0.6_ PSCs aged at 85 °C. Reproduced with permission.^[^ ^95^ ^]^ Copyright 2023, Elsevier. d) Long‐term stability of FASnI_3_ PSCs stored in an N_2_ environment. Reproduced with permission.^[^ ^99^ ^]^ Copyright 2021, Wiley‐VCH. e) Long‐term stability of unencapsulated a Sn–Pb PSC under continuous light illumination in an N_2_ environment. Reproduced with permission.^[^ ^52^ ^]^ Copyright 2022, Nature Publishing Group.

The MA cation in Sn‐containing perovskite structures has presented challenges due to its volatile nature when exposed to heat and light. To address this issue while maintaining high efficiency, our research group^[^ ^51^ ^]^ proposed a bilayer interdiffusion growth (BIG) process for fabricating stable and efficient Sn‐Pb perovskite films with reduced MA content. By employing this method, the FA_0.85_MA_0.1_Cs_0.05_Sn_0.5_Pb_0.5_I_3_ films with significantly enhanced thermal stability were synthesized. TPD‐MS analysis revealed that the thermal decomposition of MA‐less (10% MA) Sn–Pb perovskite occurred at much higher temperatures than conventional Sn–Pb perovskite with 40% MA (Figure 3e,f), indicating that the reduction in MA content contributed to enhanced thermal stability in the Sn–Pb perovskites. As a result, the corresponding Sn–Pb PSCs with reduced MA content showed improved stability than control devices when aged at 85 °C (**Figure** **7**a). Additionally, due to the reduced MA content, the photovoltaic device fabricated using this approach exhibited a remarkable retention of 92% of its initial efficiency after undergoing MPP tracking for 450 h. Thus far, pure FASnI_3_ composition has proven more advantageous in Sn‐perovskites owing to its enhanced thermal and light stability. This is exemplified by the work of Seo et al.^[^ ^48^ ^]^ which showcased that the FA‐based Sn PSC retained 83% of its initial PCE even after continuous operation for 1000 h (Figure 7b).

Another approach to reducing MA content in Sn‐containing perovskites involves replacing MA cations with Cs cations. Similar to pure‐Pb PSCs, FA/Cs‐based Sn‐containing PSCs also exhibit improved thermal and operational stability. A study conducted by Chen et al.^[^ ^94^ ^]^ specifically demonstrated the enhanced stability achieved by eliminating MA cations in FA_0.75_Cs_0.25_Pb_0.5_Sn_0.5_I_3_ PSCs. The MA‐free perovskite device maintained ≈95.1% of its initial efficiency after 500 h of heating at 85 °C, and retained 98.7% of its origin PCE after 150 h of MPP tracking under continuous light illumination. These findings indicate that the complete elimination of MA cations can substantially enhance the operational stability of Sn‐Pb perovskite solar cells. In addition, fully replacing A‐site cations with Cs cations can enhance the thermal stability of Sn‐containing PSCs. Yang et al.^[^ ^95^ ^]^ demonstrated that the all‐inorganic Sn‐Pb PSCs based on the CsPb_0.4_Sn_0.6_I_2.4_Br_0.6_ absorber layer prepared using lead acetate precursor exhibited superior thermal stability at 85 °C, maintaining 72% of its original efficiency after being subjected to heat for 700 h (Figure 7c).

#### 3.2.2 Large Cations

The employment of A‐cations with larger ionic radii (>253 pm), such as phenethylammonium (PEA) and guanidinium (GA), has proven to be a successful strategy in improving the long‐term stability and reliability of Sn‐containing PSCs.^[^ ^52^, ^96^ ^]^ Replacing A‐site cations with these larger cations leads to a reduction in the dimensionality of the perovskite structure from a three‐dimensional (3D) configuration to a mixture of two‐dimensional (2D) and 3D perovskite phases. The presence of 2D phases on the surface of the perovskite grains acts as a barrier, effectively hindering the ingress of oxygen and water molecules and preventing the egress of decomposition products.^[^ ^97^, ^98^ ^]^ He et al.^[^ ^99^ ^]^ investigated the utilization of 4‐fluoro‐phenethylammonium bromide (FPEABr) as a replacement for formamidinium iodide (FAI) in forming a 2D/3D heterogeneous tin‐based perovskite absorber. By introducing a 2D tin‐perovskite capping layer based on FPEA^+^, a conducive reducing environment was established for the susceptible 3D FASnI_3_ grains. Consequently, the oxidation of Sn^2+^ to Sn^4+^ species was effectively suppressed. With a 2D/3D structure, the encapsulated device exhibited enhanced stability when stored in N_2_, outperforming the pristine device (Figure 7d). Similarly, Zhu et al.^[^ ^52^ ^]^ demonstrated superior light stability for Sn–Pb PSCs by combining large cations, PEA^+^ and GA^+^, in perovskite precursor. The corresponding device remained 82% of its maximum efficiency for 1 830 h under 1 sun illumination in an N_2_ environment (Figure 7e).

#### 3.2.3 Halide Engineering

As previously mentioned, the degradation of Sn‐containing PSCs can be intrinsically caused by the oxidation of iodide ions. One approach to address this issue is by introducing anions (e.g., Br^−^ and Cl^−^) with a higher electronegativity than I^−^ to reduce the formation of I_2_ in Sn‐containing PSCs. This substitution is effective because halides with a higher electronegativity exhibit higher bonding strength with Pb and enhanced stability against oxidation. Moreover, partially replacing I^−^ with the smaller halides leads to a reduction in the dimensions of the perovskite unit cell. The more compact lattice structure may hinder the ingress of oxygen and moisture, thereby improving the stability of Sn‐containing PSCs.^[^ ^100^ ^]^ While incorporating smaller halides like fluorine (F^−^) and Cl^−^ into the perovskite crystal lattice is challenging due to their size, the addition of compounds such as SnF_2_ or SnCl_2_ as additives can help suppress the formation of Sn^4+^,^[^ ^40^ ^]^ which acts as a trigger for halogen formation. By inhibiting Sn^4+^ formation, these additives contribute to the stability of Sn‐containing PSCs.

The addition of pseudohalides, specifically thiocyanates (SCN^−^) and tetrafluoroborate (BF_4_^−^), has proven to be a successful strategy for enhancing the stability of Sn‐containing PSCs.^[^ ^101^, ^102^ ^]^ Huang et al.^[^ ^102^ ^]^ reported that the incorporation of octylammonium tetrafluoroborate (OABF_4_) as an additive demonstrated a significant improvement in the stability of Sn‐Pb PSCs. This improvement was attributed to the strong binding affinity between the BF_4_^−^ anion and Sn^2+^/Pb^2+^ ions within the perovskite lattice, effectively reducing the concentration of I vacancies and suppressing the formation of I_2_. Consequently, the device incorporating OABF_4_ exhibited exceptional stability, retaining 88% of its initial efficiency after undergoing MPP tracking for 1000 h.

### 3.3 Interface Passivation

Enhancing the top interface properties of the perovskite absorber layer becomes increasingly vital when dealing with Sn‐containing PSCs due to the easy oxidation of Sn^2+^ at the surface and the inferior film quality caused by rapid crystallization.^[^ ^45^, ^103^ ^]^ Many strategies have been proposed to passivate the top surface of Sn‐containing PSCs, including 2D perovskites, small organic molecules, and other Lewis base molecules.

**Figure 8:**
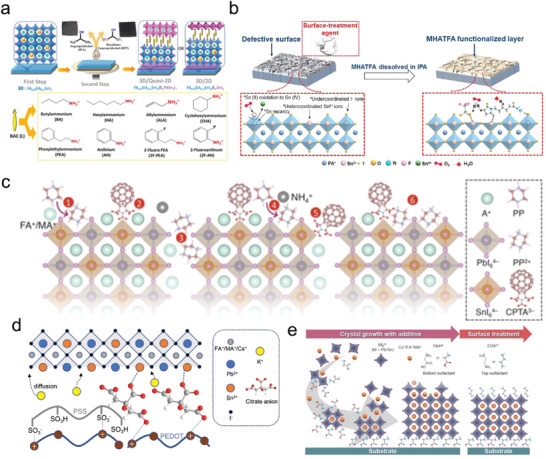
Interface passivation to improve stability. a) Schematic diagram of the top surface passivation using different organic cations. Reproduced with permission under the terms of the CC‐BY Creative Commons Attribution‐NonCommercial‐NoDerivatives 4.0 International license (https://creativecommons.org/licenses/by‐nc‐nd/4.0/).^[^ ^105^ ^]^ Copyright 2022, American Chemical Society. b) Schematic illustration of surface reconstruction strategy for Sn PSCs using MHATFA. Reproduced with permission.^[^ ^108^ ^]^ Copyright 2022, American Chemical Society. c) Schematic working mechanism of surface modification of Sn–Pb PSCs using PP and CPTA. Reproduced with permission.^[^ ^111^ ^]^ Copyright 2023, Wiley‐VCH. d) Schematic diagram of the buried interface passivation of Sn–Pb PSCs using PC. Reproduced with permission.^[^ ^114^ ^]^ Copyright 2023, Wiley‐VCH. e) Schematic illustration of dual‐surface passivation for Sn–Pb PSCs using EDAI_2_ and GlyHCl. Reproduced with permission.^[^ ^115^ ^]^ Copyright 2022, Royal Society of Chemistry.

The formation of a 2D perovskite capping layer on top of 3D perovskite can hinder the ingress of oxygen and water molecules, significantly improving the stability of Sn‐containing PSCs. He et al.^[^ ^104^ ^]^ applied an ultrathin layer of PEABr to the surface of FASnI_3_ and showed enhanced light stability in devices with the PEABr surface treatment than the control device. This improvement was attributed to the low‐dimension perovskite layer on the top surface of the perovskite films, which effectively inhibited the oxidation of Sn^2+^ ions, and reduced the density of structural defects. Further, Diau et al.^[^ ^105^ ^]^ introduced a novel sequential deposition method utilizing a solution processing technique with hexafluoro‐2‐propanol as the solvent. This method involved depositing eight bulky ammonium cations onto the surface of the Sn perovskite layer to create a hybrid 3D/quasi‐2D layer (**Figure** **8**a), which provides additional protection to the Sn perovskite surface by preventing moisture penetration. Notably, among the eight tested bulky ammonium cations, the device incorporating anilinium (AN) without additional encapsulation demonstrated exceptional stability when exposed to ambient air for 150 h.

In addition to 2D perovskites, some small organic molecules can be coated on top of the perovskite layer to improve the stability of Sn‐containing PSCs. Han et al.^[^ ^106^ ^]^ introduced a surface modification approach for FASnI_3_ perovskite by spin‐coating a solution containing n‐propylammonium iodide (PAI), an organic salt, prior to the annealing of the absorber layer. In contrast to the formation of a typical 2D perovskite structure, the inclusion of PAI facilitated a templated growth mechanism in FASnI_3_ crystals through the reconstruction of the intermediate phase. This templated growth led to a highly crystallized perovskite film with a reduced density of trap states. As a result of these improvements, the device demonstrated remarkable stability, exhibiting a retention of over 95% of its initial efficiency even after 1000 h of operation at MPP. To eliminate the Sn^4+^ defects on the surface of Sn perovskites, Zhou et al.^[^ ^107^ ^]^ proposed a chemo‐thermal surface dedoping technique for improving stability. In this method, formamidinium hydrochloride (FACl) was thermally evaporated and allowed to interact with SnI_4_ on the surface, forming a complex SnI_4_·*x*FACl, which exhibited a lower volatilization temperature, facilitating its easy removal during the subsequent thermal process. Consequently, the device incorporating this dedoping strategy demonstrated enhanced stability compared to the control device. Recently, Yin et al.^[^ ^108^ ^]^ proposed a surface‐reconstruction technique for enhancing the performance and stability of FASnI_3_ PSCs by employing post‐treatment with 6‐maleimidohexanehydrazide trifluoroacetate salt (MHATFA). The MHATFA compound was found to effectively address superficial Sn^4+^ defects through the reductive hydrazide group. Additionally, trifluoroacetate (TFA) anions in MHATFA exhibited strong coordination with surface Sn^2+^ ions, resulting in the passivation of donor‐type defects such as undercoordinated Sn, Sn interstitials, and Sn–I antisites (Figure 8b). Furthermore, MHATFA facilitated the immobilization of I^−^ ions through hydrogen bonding interactions with the iodine atoms. As a result, the passivated device demonstrated superior light stability compared to the non‐passivated device, retaining 76.4% of its initial efficiency even after continuous light exposure for 1000 h.

Due to the Lewis acid nature of Sn(II) compounds, a Lewis base can effectively coordinate with them and thus improve their stability against oxidation. Hayase et al.^[^ ^109^ ^]^ introduced an ethylenediamine Lewis base post‐treatment technique to mitigate the oxidation of Sn^2+^ and enhance the stabilization of undercoordinated Sn on the surface of Sn perovskites. Yin et al.^[^ ^110^ ^]^ presented a successful passivation approach using thiosemicarbazide to address surface defects arising from undercoordinated Sn and Sn^2+^ oxidation in CsSnI_3_ perovskites. This passivation strategy resulted in a significantly extended device lifetime, with the treated device retaining over 90% of its initial PCE even after continuous illumination for 500 h. Recently, Wakamiya et al.^[^ ^111^ ^]^ proposed a synergistic surface modification strategy that involved the combined use of piperazine (PP) and C_60_ pyrrolidine tris‐acid (CPTA), as depicted in Figure 8c. In this approach, PP underwent protonation through a reaction with the organic cations present on the surface of the perovskite film, thereby effectively passivating the A‐site vacancies. Simultaneously, the carboxyl groups in CPTA formed strong coordination bonds with the metal cations, consequently suppressing the oxidation of Sn^2+^. As a result of this dual modification, the treated solar cells exhibited exceptional stability, with unencapsulated cells retaining 96% of their initial efficiency even after storage for over 2000 h under N_2_ conditions.

Apart from the top surface passivation, the buried interface passivation also plays an important role in achieving the long‐term stability of Sn‐containing PSCs. Chen et al.^[^ ^112^ ^]^ investigated the utilization of poly[tetraphenylethene 3,3′‐(((2,2‐diphenylethene‐1,1‐diyl)bis(4,1‐phenylene))bis(oxy))bis(N,N‐dimethylpropan‐1‐amine)tetraphenylethene] (PTN‐Br) as a hole transport material positioned between FASnI_3_ and PEDOT:PSS. This approach effectively mitigated the presence of trap states, resulting in enhanced UV stability. Furthermore, Tai et al.^[^ ^113^ ^]^ presented a buried interface passivation technique aimed at controlling the buried interface between NiO*_x_* and FAPb_0.5_Sn_0.5_I_3_ perovskites. This passivation approach involved the utilization of 4‐hydroxyphenethyl ammonium bromine in reducing surface defects within the NiO*_x_* hole transport layer and enhancing the quality of the perovskite film. Consequently, the passivated device demonstrated enhanced stability, retaining 80% of its initial PCE for a continuous light soaking exceeding 800 h. Recently, a study conducted by our research group^[^ ^114^ ^]^ revealed the presence of a defective buried interface in Sn‐Pb perovskites, which was attributed to the susceptibility of Sn^2+^ to oxidation and the strong acidity of PEDOT:PSS. To mitigate these challenges, we introduced potassium citrate into PEDOT:PSS to neutralize its acidity and thereby enhance interface stability. Additionally, the citrate ions coordinated with Sn^2+^ species, effectively preventing their oxidation at the buried surface, as depicted in Figure 8d. Consequently, the passivated device demonstrated improved light stability compared to the non‐passivated counterpart.

Based on the discussion above, it is evident that achieving passivation at both the top and buried interfaces is crucial for enhancing the stability of Sn‐containing PSCs. Wakamiya et al.^[^ ^115^ ^]^ proposed a dual interface passivation strategy for Sn–Pb perovskites, utilizing ethylenediammonium diiodide (EDAI_2_) and glycine hydrochloride (GlyHCl), as depicted in Figure 8e. The EDAI_2_ passivation on the top surface effectively reduced trap densities, thus improving the surface quality. On the other hand, the GlyHCl treatment targeted the passivation of recombination centers in the buried region. The combined effect of these two passivation approaches synergistically enhanced the stability of Sn–Pb PSCs.

### 3.4 Device Architecture Innovations

In the context of regular‐structured Sn‐containing PSCs, the commonly employed hole transport layer (HTL), Spiro‐OMeTAD, has been found to contribute to the instability of such devices. To address this issue, Kanatzidis et al.^[^ ^116^ ^]^ synthesized a novel HTL incorporating 4,8‐di(thiophen‐2‐yl)benzo[1,2‐b:4,5‐b']dithiophene (BDT) as the central unit, along with tetra‐4,4′‐dimethoxytriphenylamine substituents. Notably, the PSC device utilizing this newly developed HTL demonstrated favorable stability, exhibiting a retention of 66.9% of its initial PCE after being stored in a nitrogen environment for ≈14 months.

**Figure 9:**
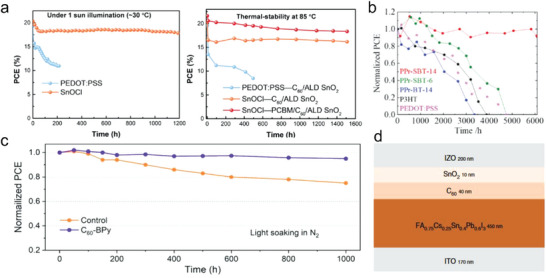
Device architecture innovations to improve stability. a) Long‐term light and thermal stability of Sn‐Pb PSCs based on SnOCl and PEDOT:PSS HTLs. Reproduced with permission.^[^ ^119^ ^]^ Copyright 2022, Wiley‐VCH. b) Long‐term stability of Sn PSCs with different HTLs stored in N_2_ environment. Reproduced with permission.^[^ ^120^ ^]^ Copyright 2023, Wiley‐VCH. c) Long‐term light stability of Sn PSCs with different ETLs. Reproduced with permission.^[^ ^122^ ^]^ Copyright 2022, Wiley‐VCH. d) Schematic device configuration of a Sn–Pb PSC. Reproduced with permission.^[^ ^123^ ^]^ Copyright 2019, Nature Publishing Group.

To date, the majority of Sn‐containing PSCs have been fabricated using an inverted structure, as it offers higher efficiency than the regular structure. However, the commonly used HTL, PEDOT:PSS, has been identified as a factor contributing to the instability of Sn‐PSCs. To address this issue, McGehee et al.^[^ ^117^ ^]^ introduced poly[bis(4‐phenyl)(2,4,6‐trimethylphenyl)amine] (PTAA) as an alternative HTL for Sn‐Pb PSCs, resulting in improved thermal stability. The PTAA‐based device demonstrated an impressive 80% retention of its initial PCE after aging for 4000 h of thermal stress at 85 °C. Another approach to eliminating the use of PEDOT:PSS as the HTL was presented by Han et al.^[^ ^118^ ^]^ They designed an HTL‐free structure for Sn‐PSCs, where the device was grown directly on a bare indium tin oxide (ITO) substrate. This configuration exhibited enhanced stability against light soaking for 40 days and thermal stress at 80 °C for 300 h. More recently, Huang et al.^[^ ^119^ ^]^ reported the utilization of a novel HTL, SnOCl, as a replacement for PEDOT:PSS in Sn‐Pb PSCs. The introduction of SnOCl as the HTL significantly enhanced the stability of Sn‐Pb PSCs, with 87% of the initial efficiency retained after 1200 h of continuous 1‐sun illumination and 85% efficiency maintained under 85 °C thermal stress for 1500 h (**Figure** **9**a). Furthermore, Diau et al.^[^ ^120^ ^]^ developed a dopant‐free pyrrolopyrrole‐based polymeric HTL for Sn perovskites. This innovative HTL exhibited remarkable long‐term stability, surpassing 6000 h when stored in a nitrogen environment (Figure 9b).

In addition to advancements in HTLs, the stability of Sn‐containing PSCs has also been improved through innovations in ETL. Ning et al.^[^ ^121^ ^]^ introduced indene‐C60 bisadduct (ICBA) as an alternative ETL to replace the commonly used PCBM. ICBA possesses a shallower energy level, effectively mitigating iodide remote doping in the ETL. This, in turn, results in enhanced stability of the Sn PSCs. To further enhance stability by modifying the interface between the perovskite and ETL, Zhu et al.^[^ ^122^ ^]^ developed a pyridine‐functionalized fullerene derivative called C_60_‐BPy as the ETL for Sn PSCs. The pyridine unit in C_60_‐BPy strongly bonds to the Sn‐exposed surface, reducing the trap density at the perovskite surface. This interface modification contributes to superior stability, as the Sn PSC device maintained over 95% of its initial efficiency even after continuous light illumination for 1000 h (Figure 9c).

To address the instability issues associated with PEDOT:PSS and metal electrodes in Sn‐Pb PSCs, McGehee et al.^[^ ^123^ ^]^ devised a stable device structure by implementing an HTL‐free configuration and employing indium zinc oxide (IZO) to replace the metal electrode (Figure 9d). The HTL‐free structure eliminated the potential reaction between the perovskite and PEDOT:PSS HTL, mitigating stability concerns. Furthermore, the utilization of an IZO electrode effectively capped the perovskite layer, thereby inhibiting the oxidation of Sn–Pb perovskites. As a result, the novel device structure demonstrated an impressive 95% retention of its initial efficiency after being subjected to 1000 h of thermal stress at 85 °C in an air environment without encapsulation. Additionally, the Sn‐Pb PSCs maintained their initial efficiency levels during the operation near the maximum power point and under continuous 0.8‐sun illumination for over 1000 h.

In the common superstrate configuration for all‐perovskite tandems, one of the challenges is that the Sn–Pb perovskite layer is typically assembled last and is easily exposed to air, leading to the degradation of the tandem devices. To overcome this setback, Tan et al.^[^ ^124^ ^]^ proposed a solution by developing a substrate‐configured tandem device structure. In this structure, the easily oxidizable Sn–Pb perovskite subcell is deposited first, followed by the interconnecting layer and front subcell, effectively creating a self‐encapsulation mechanism. This arrangement acts as a barrier, preventing oxygen ingress into the Sn‐Pb perovskite absorber layer and enhancing the stability of tandem devices.

### 3.5 Encapsulation Engineering

The primary obstacle in commercializing Sn‐containing PSCs is their susceptibility to moisture and oxygen, which significantly impacts their stability. To overcome this challenge, it is crucial to focus on developing appropriate encapsulation materials and processes to effectively preserve the stability of Sn‐containing perovskites. The efficiency of the protective encapsulant is determined by two key parameters: the oxygen transmission rate (OTR) and the water vapor transmission rate (WVTR).^[^ ^125^ ^]^ When evaluating the suitability of a material for use as an encapsulant, the WVTR holds greater significance than the OTR. This is because water vapor molecules are smaller than oxygen molecules, rendering their containment more difficult. Therefore, the ability of an encapsulant to effectively impede the passage of water vapor is of paramount importance in ensuring the long‐term stability of the system.

The glue‐based encapsulation technique is frequently employed as a protective strategy for Sn‐containing perovskite materials. This method typically entails the use of a thin glass coverslip, which is affixed to the solar cells using a UV‐curable adhesive. In many cases, UV epoxy materials originally developed for encapsulating organic electronic technologies have been adapted for use in PSCs.^[^ ^126^ ^]^ These epoxy materials typically exhibit WVTR ranging from 0.7 to 0.94 g m^−2^ d^−1^.^[^ ^127^ ^]^ Therefore, due to their relatively high WVTR values, this type of encapsulation is more suitable for short‐term sample protection, such as conducting *J–V* tests in atmospheres outside of a glovebox environment. In the pursuit of long‐term sample protection, Khenkin et al.^[^ ^128^ ^]^ proposed a lamination‐based glass‐to‐glass encapsulation approach for PSCs. This encapsulation strategy involved the utilization of a butyl rubber edge sealant, e.g., polyisobutene (PIB), and a polyolefin film as the encapsulant, which are commonly employed in commercial thin‐film solar modules. When combined with a desiccant, the butyl rubber edge sealant exhibits a low water WVTR, thus providing superior protection against moisture and oxygen ingress into the device. As a result of implementing this encapsulation method, a PSC demonstrated minimal efficiency loss even after undergoing a 6‐month outdoor stability test. This encapsulation scheme could be potentially used in Sn‐containing PSCs, but its OTR needs further evaluation.

## 4 Conclusions and Outlook

**Figure 10:**
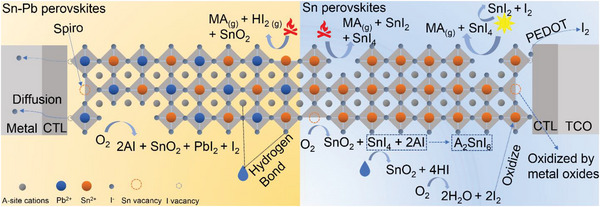
Schematic diagram of degradation mechanisms of Sn‐containing perovskites.

This review article critically overviews the degradation mechanisms of Sn‐containing perovskites and discusses various strategies to mitigate the degradation. **Figure** **10** summarizes the major degradation pathways in Sn‐containing perovskites. The primary challenge in achieving long‐term stability for Sn‐containing perovskites arises from the facile oxidation of Sn^2+^, an inherent characteristic of these materials. This oxidation process hinders the attainment of sustained durability in Sn‐containing perovskites. Moreover, the degradation of perovskites in operational conditions leads to the relatively easy formation of native I_2_, further exaggerating Sn^2+^ oxidation and deteriorating Sn‐containing perovskites. In addition to these intrinsic factors, Sn‐containing perovskites exhibit instability when exposed to extrinsic factors, including light, heat, moisture, oxygen, and their combinations. These environmental stressors induce the degradation of Sn‐containing perovskites, involving various mechanisms such as the decomposition of A‐site cations, I_2_ formation, oxidation of SnI_2_, and even the decomposition of common impurity SnI_4_.

To combat the degradation caused by both intrinsic and extrinsic factors, several strategies have been proposed and summarized. These strategies include additive engineering, which involves the incorporation of specific antioxidants and reducing additives to enhance the stability of Sn‐containing perovskites. A‐site and X‐site compositional engineering is another approach that focuses on optimizing the perovskite composition to improve the durability of these materials. Additionally, interface passivation and device structure innovation also play critical roles in enhancing the device stability of Sn‐containing perovskites. Lastly, for the practical operation of Sn‐containing PSCs under real‐world conditions, encapsulation engineering techniques have been explored to provide a protective barrier and shield Sn‐containing perovskites from the detrimental effects of external factors.

Despite significant advancements, the commercial development of Sn‐containing perovskites is still hindered by their inherent instability. To enhance the stability of these materials, the following outlooks can be pursued:

1) Eliminating SnI_4_ formation at every stage of the life cycle of Sn‐containing PSCs. SnI_4_ can be present throughout the entire process, including the source materials, perovskite precursor preparation, processing of the perovskite film, the entire device fabrication, and complete devices under operation. Although certain sacrificial reducing agents like Sn powders can effectively mitigate the Sn^4+^ content in the precursor, Sn^4+^ can still form during the fabrication and encapsulation. Therefore, more potent and advanced antioxidation strategies are required to suppress the formation of Sn^4+^ in Sn‐containing perovskites. This requires in‐depth studies of Sn‐perovskite single crystals to elucidate the origins and evolution of Sn^4+^ defects on the surface and in the bulk of Sn‐perovskite crystals and to develop mitigating strategies.
2) Inhibiting I_2_ formation in Sn‐containing perovskites. I_2_ formation arises from various processes, including the photodecomposition of SnI_2_ and the redox reaction of I^−^/I_2_ under illumination, heat, and electric bias. As mentioned earlier, the presence of I_2_ in Sn‐containing perovskites further oxidizes Sn^2+^, contributing to perovskite degradation. To address this issue, one approach involves in‐situ neutralization of I_2_ by incorporating sacrificial reductants, ionic liquids, and appropriate Lewis molecules into Sn‐containing perovskites. Increasing grain size and reducing grain boundaries also contribute to the suppression of I_2_ formation.
3) Interface engineering is a highly targeted strategy to prevent degradation as it is the primary location where degradation tends to occur. Currently, most interface passivation techniques for Sn‐containing PSCs are adopted from Pb‐based PSCs. However, certain processes involved in these passivation strategies are unsuitable for Sn‐containing PSCs. For instance, solution post‐treatments with isopropyl alcohol as a solvent can be detrimental to Sn‐containing perovskites. Consequently, future research should prioritize the selection of appropriate solvents or explore dry vapor‐based passivation techniques like thermal evaporation and chemical vapor surface treatment. Moreover, the unstable HTL layer, e.g., PEDOT:PSS, is another critical factor limiting the long‐term stability of Sn‐containing PSCs. To enhance the stability of Sn‐containing PSCs, it is important to design novel HTL that can stabilize the buried interface effectively. Lastly, metal contacts like Ag tend to react with halides present in the perovskite absorber layer, negatively impacting the stability of PSCs. A promising research direction for achieving long‐term stability in Sn‐containing PSCs is to explore transparent conducting oxide (TCO) electrodes. These TCO electrodes exhibit better resistance to halide diffusion and corrosion. The compactness of TCO capping layer can also prevent the ingress of oxygen and moisture. Therefore, investigating TCO capping electrodes holds great potential as a superior alternative to traditional metal electrodes.
4) Enhancing encapsulation technology. Encapsulation techniques such as UV‐curable adhesive and glass‐to‐glass encapsulation, borrowed from other photovoltaic technologies, have shown promise in protecting Sn‐containing PSCs against moisture and oxygen in laboratory settings. However, such encapsulation methods may prove insufficient for outdoor testing. Advanced packaging approaches are being explored, such as glass‐to‐glass encapsulation with ethylene‐vinyl acetate and rubber edge sealing. However, these methods typically require processing temperatures between 100 and 150 °C, which can be detrimental to Sn‐containing perovskites. Therefore, developing robust and low‐temperature encapsulation technologies represents a future direction to enhance the long‐term stability of Sn‐containing PSCs.

In conclusion, the durability of Sn‐containing PSCs has yet to be demonstrated, primarily because of the defective surfaces of polycrystalline perovskite thin film. The decent stability of Sn‐perovskite single crystals shows the promise of achieving stable Sn‐containing perovskite thin films if their interfaces and grain boundaries can be fully stabilized. With increasing research efforts dedicated to exploring the intrinsic stability of Sn‐containing perovskite materials, future advances in a variety of material processing and device fabrication techniques, and innovation in device architecture, the durability issue of Sn‐containing PSCs would be alleviated or resolved. We maintain a cautiously optimistic outlook for the future of Sn‐containing PSCs and believe that the immense potential of this promising PV technology will be unleashed despite its inherent instability, leading to tangible benefits for society and the environment.

## Conflict of Interest

The authors declare no conflict of interest.
